# Phenotypic and Genotypic Characterization with MALDI-TOF-MS Based Identification of *Staphylococcus* spp. Isolated from Mobile Phones with their Antibiotic Susceptibility, Biofilm Formation, and Adhesion Properties

**DOI:** 10.3390/ijerph17113761

**Published:** 2020-05-26

**Authors:** Emira Noumi, Abderrahmen Merghni, Mousa Alreshidi, Rosa Del Campo, Mohd Adnan, Ons Haddad, Vincenzo De Feo, Mejdi Snoussi

**Affiliations:** 1 Department of Biology, College of Science, University of Ha’il, Hail 2440, Saudi Arabia; eb.noumi@uoh.edu.sa (E.N.); mo.alreshidi@uoh.edu.sa (M.A.); mo.adnan@uoh.edu.sa (M.A.); 2 Laboratory of Bioressources: Integrative Biology and Recovery, High Institute of Biotechnology-University of Monastir, Monastir 5000, Tunisia; 3 Laboratory of Antimicrobial Resistance (LR99ES09), Faculty of Medicine of Tunis, University of Tunis El Manar, Tunis 1007,Tunisia; abderrahmen.merghni@fmt.utm.tn; 4 Servicio de Microbiología, Instituto Ramón y Cajal de Investigación Sanitaria 14 (IRYCIS), Hospital Universitario Ramón y Cajal, Carretera de Colmenar, 28034 Madrid, Spain; rosa.campo@salud.madrid.org; 5 Laboratoire de Microbiologie, CHU Fattouma Bourguiba de Monastir, Monastir 5000, Tunisia; onshadad@gmail.com; 6 Department of Pharmacy, University of Salerno, Via Giovanni Paolo II, 132, Fisciano, 18, 84084 Salerno, Italy; 7 Laboratory of Genetics, Biodiversity and Valorisation of Bioressources, High Institute of Biotechnology-University of Monastir, Monastir 5000, Tunisia

**Keywords:** mobile phone, MRSA, *Staphylococcus*, virulence genes, antibiotic resistance, biofilm, MALDI-TOF-MS

## Abstract

Cell phones, smartphones, and tablets are extensively used in social and professional life, so they are frequently exposed to bacteria. The main goal of the present work was to isolate and characterize *Staphylococci* strains from students’ cell phone mobiles. Subsequently, 24 *Staphylococci* strains were tested against a wide range of antibiotics, for the distribution of some virulence-related genes and their ability to form biofilm. *Staphylococcus* spp. were cultured from all studied devices on chromogenic medium and identified using the matrix-assisted laser desorption/ionization (MALDI), time-of-flight (TOF) mass spectrometry (MS) technique (MALDI-TOF-MS). The results obtained showed that *S. aureus* was the dominant species (19 strains, 79.1%), followed by *S. warneri* (3 strains, 12.5%), and *S. haemolyticus* (2 strains, 8.3%). Isolated strains showed high percentages of hydrolytic enzymes production, resistance to many tested antibiotics, and 37.5% expressed the *mecA* gene. The tested strains were highly adhesive to polystyrene and glass and expressed implicated *icaA* (62.5%) and *icaD* (66.6%) genes. All *Staphylococcus* spp. strains tested were found to possess proteases and the α-hemolysin gene. Our results highlighted the importance of mobile phones as a great source of *Staphylococcus* spp., and these species were found to be resistant to many antibiotics with multiple antibiotic resistance (MAR) index ranging from (0.444) to (0.812). Most of the studied strains are able to form biofilm and expressed many virulence genes. Phylogenetic analysis based on the phenotypic and genetic characters highlighted the phenotypic and genetic heterogeneity of the *S. aureus* population studied. Further analyses are needed to elucidate the human health risks associated with the identified *Staphylococci* strains.

## 1. Introduction

In Tunisia, the first experimental communication made by the global system for mobile telecommunication group was done in 1991 [1]. In fact, more than 77% of the world’s population owns a cell phone now [2]. This device has become one of the most indispensable accessories of professional and social life. Its use is considered as an essential part of our everyday professional as well as personal life [3,4]. Nowadays, the cell phone is an essential commodity and represents a health hazard device, because it is often maintained close to human face, ears, hands, and lips [5]. As a result, the cell phone mobiles are a potentially niche of microorganisms that can be dangerous for human health [6]. In fact, the colonization of mobile phone and smartphones by Gram-positive and Gram-negative bacterial strains belonging to *Micrococcus*, *Staphylococcus*, *Acinetobacter*, *Klebsiella*, *Escherichia*, *Streptococcus*, and *Pseudomonas* genera has been previously reported [5,6,7,8]. It has been also demonstrated that these devices used in hospital environments are frequently associated with multidrug-resistant and pathogenic bacteria [9,10,11].

*Staphylococci* constitute normal commensal flora of humans and many animals but are considered to be the leading cause of hospital and nosocomial infections [7]. The high pathogenicity is related to their ability to induce resistance against a wide range of antibiotics and adapt to the changes to a variety of environmental conditions [12]. It has been shown that the adaptation processes of *Staphylococcus aureus (S. aureus)* occur through significant changes in protein composition and metabolomic profiles that allow them to survive and colonize different biotic and abiotic surfaces [13,14,15]. These changes in proteome and metabolome could be explained by the changes in cell wall thickness, cell size, and ultimately colony size on culture plates [16].

*Staphylococcus* strains are known to be frequently isolated from biofilm developed on various medical devices [17] and mobile phones [18]. The pathogenicity of these bacteria is related to their ability to adhere to host cell-tissue promoted by the release of toxins [19]. In fact, *S. aureus* produces several hydrolytic enzymes, such as serine protease, cysteine protease, lipase enzymes, and multiple toxins, acting immediately in the area of infection [20,21].

In order to assess the hygiene applied during the use of cell phones in hospital and public environments, the adhesive capacities and the antibiotics resistance of this pathogen must be evaluated [22]. The aim of the current study was to isolate, identify, and characterize *Staphylococcus* spp. strains from students’ cell phones from a teaching Institute in Monastir (Tunisia). We also evaluated the susceptibility of 24 isolates to antibiotics, virulence features, and biofilm formation capacity on materials used in mobile and smartphones industry, i.e., mostly plastic, glass, silicon, and aluminum. The distribution of some virulence-related genes encoding resistance to methicillin, protease (*ssp*A, *ssp*B), lipase (*geh*), α-hemolysin (*hla*), and adhesion properties (*ica*A, *ica*D, *cna*, *fnb*A) in the genome of all *S. aureus*, *S. haemolyticus*, and *S. warneri* isolates was also investigated.

## 2. Results

### 2.1. Staphylococcus spp. Morphotypes on CSA

**Figure 1:**
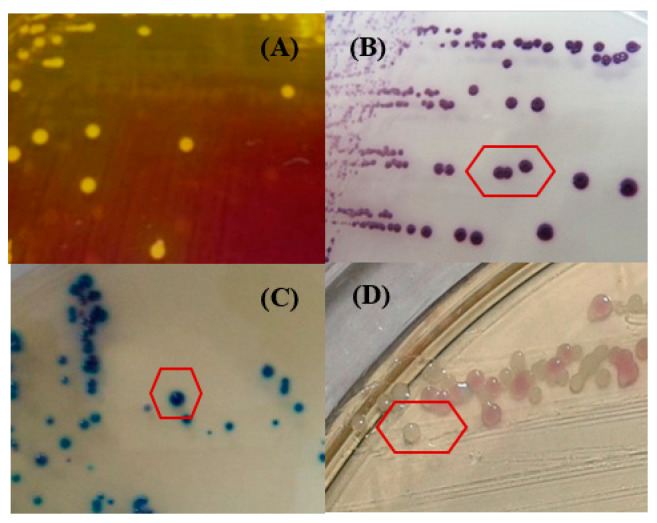
*Staphylococcus* spp. morphotypes obtained on Chapman agar (**A**) and CHROM^TM^ Staph agar (**B)**: *S. aureus* ATCC 43300; (**C**) *S. haemolyticus*; (**D**) *S. warneri*).

On the two tested media, colonies were about 2 to 3 mm in diameter after 24 h of incubation. The results on Chapman medium were represented by three different morphotypes: 29.5% yellow colonies (Figure 1A), 6.1% pink colonies, and 64.28% white colonies.

On CSA, three morphotypes characterizing the *Staphylococcus* species were noted (Table 1): 35.67% mauve colonies (*S. aureus*: Figure 1B) as compared to *S. aureus* ATCC 43,300 reference strain, 29.14% light blue colonies (*S. haemolyticus*: Figure 1C), and 35.17% white colonies (*S. warneri*: Figure 1D).

### 2.2. Species Identification with MALDI-TOF-MS and Hydrolytic Enzymes Production

Twenty-four bacterial isolates were confirmed belonging to the *Staphylococcus* genus using MALDI-TOF-MS technique. Three distinct *Staphylococci* species were identified: *S. aureus* (*n* = 19), *S. warneri* (*n* = 3), and *S. haemolyticus* (*n* = 2). The detection of coagulase allows the differentiation of species of the genus *Staphylococcus*. In fact, 43.68% of the isolated strains were coagulase positive. On DNA agar, 64.85% of the strains resulted positive to DNAase enzyme. In addition, most strains (90.26%) were positive for catalase. Most tested strains produced several hydrolytic exoenzymes like amylase (70.83%), caseinase, and gelatinase (45.83%) with significant activity of the enzyme lecithinase (83.33%). Lipase secretion was low (29.16%) compared to other enzymes. On the other hand, 50% of the strains tested were alpha hemolytic and 29.16% were β-hemolytic (Table 2).

### 2.3. Susceptibility to Antibiotics and Detection of mecA Gene

Table 3 summarizes the results of the susceptibility test of the isolates included in this study. The antimicrobials used in this study are those recommended by the Committee of the French Society of the Antibiogram (2017).

Comparing our results with the standard limits, we noted that the strains showed a strong resistance to almost all antibiotics tested belonging to different classes including ceftazidime, cefotaxime and penicillin G (100%); cefoxitin and ticarcilin (95.83%); tetracycline (87.5%); erythromycin (79.16%), tobramycin (79.16%); sulfamethoxazole–trimethoprim (72.72%); rifampicin (RA) (66.66%); fusidic acid (62.5%); gentamicin (45.83%), and amikacin (33.33%). Nevertheless, the tested *Staphylococcus* spp. strains were sensitive to chloramphenicol, kanamycin, ofloxacin, norfloxacin, and levofloxacin (percentage not exceed 25%).

It is important to mention that twelve strains were found to be resistant to cefoxitin according to the disk diffusion method. These results were correlated to the results obtained by PCR amplification of the *mec*A gene, responsible for resistance to methicillin. Nine isolates (26M, 59B, 65C, 66B, 68T, 74M, 78T, 7T, and 39B) expressed this gene showing a band size of 310bp (Table 3).

Krumperman [23] introduced multiple antibiotic resistant (MAR) index analysis in 1983 [23]. In fact, the calculated MARI index values were ranging from 0.444 to 0.812 for the 16 *S. aureus* strains tested. Interestingly, the *S. aureus* strains (68T) was resistant to thirteen antibiotics tested with an MAR index value about 0.812. The MARI values ranged from 0.529 to 0.777 for the *S. haemolyticus* strains and from 0.666 to 0.722 for the two *S. warneri* strains.

### 2.4. Adhesive Properties

**Figure 2:**
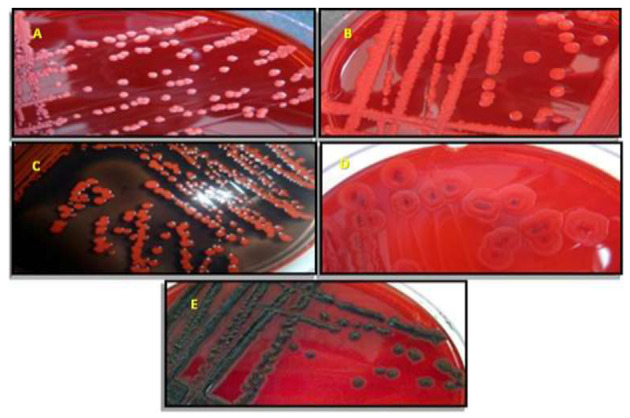
Different morphotypes obtained on Congo red agar based on the color obtained. (**A**) Pink, (**B**) red, (**C**) Bordeaux, (**D**) red colonies with dark center, and (**E**) black colonies.

The capacity of *Staphylococcus* strains to adhere to mobile phones was estimated both qualitatively and quantitatively. Five morphotypes were defined according the color scale visualized on Conge red agar: non-slime producing *Staphylococcus* spp. strains are characterized by pink, red, red with black center, and Bordeaux colonies, while black morphotype characterizing slime positive bacteria (Figure 2).

The main results showed that a strain of *S. aureus* (12C black on CRA) exhibited high biofilm formation on polystyrene (OD = 1.34 ± 0.18) and glass (1.09 ± 0.05) compared to the reference strain *S. aureus* ATCC 43,300 on polystyrene (OD = 1.89 ± 0.13) and glass (1.48 ± 0.15). Amongst all tested isolates, four (16.66%) displayed positive and variable phenotype on CRA plates, indicating slime production (Table 4).

Qualitative evaluation of biofilm formation potential on glass tubes showed that 58.33% of *Staphylococcus* isolates were strongly adherent (noted +++) and 29.16% were moderately adherent (noted ++) to this material. Furthermore, the result from 1% crystal violet (CV) staining assay showed that of all isolates tested, only three *S. aureus* isolates (12C, 67M, and 76C) and one *S. haemolyticus* strain (39B) were found to be highly adherent to polystyrene showing OD_570_ > 1. The strains 12C, 56M, and 67M of *S. aureus* were highly biofilm forming on glass strips (noted “H”) and all other strains were moderately adherent (noted “M”) to glass. Two strains of *S. aureus* (12C and 67M) were highly adherent to polystyrene and glass. All these data are summarized in Table 4.

### 2.5. Detection of Biofilm, Exoenzymes, and Haemolysin Related Genes

According to our results, ten *S. aureus* strains (52.6%) were positive for both *icaA* (amplifying a specific 198pb-band) and *icaD* (amplifying a specific 188pb-band) genes encoding the intracellular adhesion A and B factors, respectively, as compared to the *S. aureus* ATCC 43,300 positive control strain for all tested genes. Among the virulence factors identified in *S. aureus* strains, the presence of genes encoding fibronectin (*fnbA*) and collagen (*cna*) was emphasized. The *can* gene encoding the collagen binding protein was detected in the genome of twelve tested bacteria (50.0%), whereas eighteen (75.0%) expressed the *fnbA* gene. Interestingly, the four biofilm related genes were detected in the genome of eight *S. aureus* strains (42.1%).

The tested *Staphylococcus* spp. strains produced several hydrolytic enzymes (Table 2). In fact, four (16.6%) and sixteen (66.6%) strains were positive for *sspA* and *sspB*, respectively. In addition, two *S. aureus* and one *S. haemolyticus* strains (12.5%) expressed both genes (Table 5). Our results revealed also that eight isolates (33.3%) harbored the *geh* gene showing a band size of 473 bp. The *hla* gene encoding hemolysin alpha was detected in nine strains (37.5%) giving a band about 201 bp.

### 2.6. Statistical Analysis

The data from this study were captured, recorded, and analyzed by SPSS 17.0 software. The non-parametric Mann–Whitney U test was used to compare biofilm production assays and the presence of genes. We tested the correlation between biofilm forming *Staphylococcus* spp. strains on glass and polystyrene material and (exoenzymes/biofilm/hemolysin) related genes (Table 6). No difference was observed in the prevalence rate of all tested genes and the ability to form a mature biofilm on both glass and polystyrene material.

**Figure 3:**
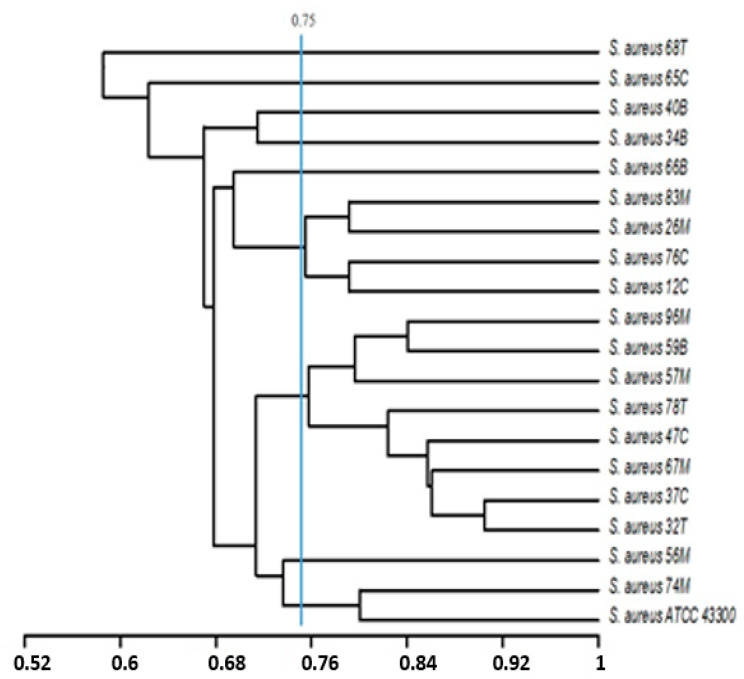
Dendrogram based on the unweighted pair group method of arithmetic averages and Jaccard’s correlation coefficient on the basis of the phenotypic characters of the *S. aureus* population (exoenzymes produced, susceptibility to antibiotics, and biofilm formation capacity). Numbers on the horizontal axis indicate the percentage of similarity.

**Figure 4:**
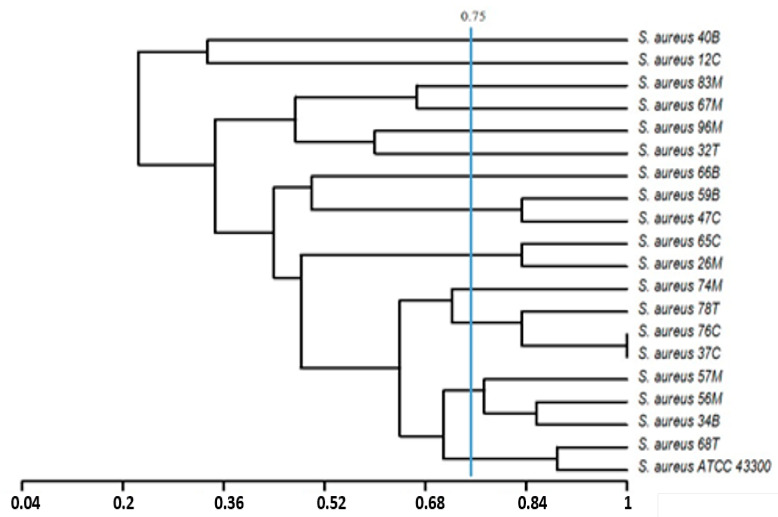
Dendrogram based on the unweighted pair group method of arithmetic averages and Jaccard’s correlation coefficient on the basis of the genetic traits of the *S. aureus* population (distribution of some virulence-related genes: *mec*A; *ssp*A, *ssp*B; *geh*; *hla*; *ica*A, *ica*D, *cna*, *fnb*A). Numbers on the horizontal axis indicate the percentage of similarity.

Dendrograms were constructed using the unweighted pair group method of arithmetic averages (UPGMA) and Jaccard’s correlation coefficient based on the phenotypic features (exoenzymes produced, susceptibility to antibiotics, and biofilm formation capacity), and/or genetic traits (distribution of some virulence-related genes: *mec*A; *ssp*A, *ssp*B; *geh*; *hla*; *ica*A, *ica*D, *cna*, *fnb*A). The phylogenetic analysis of the obtained patterns based on both phenotypic and genetic characters has shown that the *S. aureus* population studied (20 isolates) exhibit high amount of heterogeneity. For a degree of similarity greater than 0.75, we founded the presence of 9 phylogroups (Figure 3) and 13 genogroups (Figure 4) highlighting the heterogeneity of the *S. aureus* population studied. 

**Figure 5:**
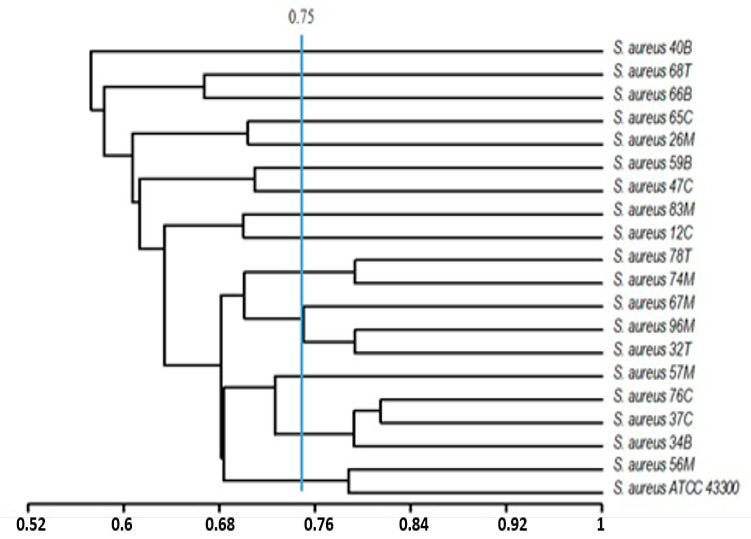
Dendrogram based on the unweighted pair group method of arithmetic averages and Jaccard’s correlation coefficient based on the phenotypic and genetic traits of the *S. aureus* population. Numbers on the horizontal axis indicate the percentage of similarity.

The high variability in the patterns obtained based on the phenotypic and genetic features clustered together (for a degree of similarity greater than 0.75, we found the presence of 14 clusters) highlighted the high diversity in the *S. aureus* population from cell phones (Figure 5).

## 3. Discussion

The misuse of smartphones, especially cell phones and the lack of safety hygiene practices, make them a potent source of pathogenic microorganisms. Furthermore, it has been demonstrated that humidity and temperature are the main environmental factors known to affect the biodiversity of microorganisms colonizing cell phones [24]. Through every phone call, there is a risk of bacterial contamination to different parts of the human body accentuated by hand-to-facial contact (lips and ears) or by hand to the surrounding surface [25,26,27,28,29]. In fact, the percentage of contaminated cell phones in the studies made by Tagore et al. [26], Bhat et al. [6], Ulger et al. [28], and Badr et al. [29] were 100%, 99%, 94.5%, and 93.7%, respectively.

Several studies from different parts in India showed that the predominant organisms isolated from contaminated cell phones are Coagulase Negative *Staphylococci* (CoNS) followed by *Staphylococcus aureus*, *Escherichia coli*, *Klebsiella pneumoniae*, *Acinetobacter* sp, *Enterococcus faecalis*, and *Pseudomonas aeruginosa* [30,31]. Additionally, other microorganisms have been reported to be the main harmful bacteria isolated from health professional’s mobile phones in a tertiary care hospital of eastern part of Bengal including *S. epidermidis*, *Micrococcus* sp., *B. subtilis*, diphtheroids, *E. faecalis*, α-hemolytic streptococci, and non-fermenter Gram-negative coccobacilli [32]. Interestingly, strains with high resistance to antibiotics, especially to methicillin, aminoglycoside, carbapenem, and Extended-spectrum beta-lactamases, or ESBLs, producing organisms were also identified [33].

Our results indicated that the majority of the tested isolates were resistant to the tested antibiotics (MARI ranging from 0.444 to 0.812) including those routinely used to treat infection due to the *S. aureus* species. The same results have previously been reported in China and Russia [34,35]. Interestingly, the tested *S. aureus* strains identified in the present study were resistant to erythromycin (79.16%). Similar results have been observed in China (97.8%) [36], United Kingdom (90%) [37], and Australia (98%) [38]. As compared to previous studies, our identified *Staphylococci* strains were highly resistant to tetracycline (87.5%), whereas it was 48% and 44% in Lebanon [39,40] and (5%) in the USA [41].

Antibiotic sensitivity patterns of the isolates showed that 21.05% of *S. aureus* were resistant to methicillin (MRSA). Different studies reported the incidence of MRSA isolated from cell phones was variable in different geographical areas like 16.9% in Mumbai [42], 52.4% in Bhabnagar [43], 52% in Turkey [28], and only 12% in Iran [44].

Clinical *S. aureus* species produces several types of secreted exoenzymes such as hemolysins and adhesins [45,46,47,48,49,50]. This bacterium is known to produce about twenty-three Staphylococcal Enterotoxins (SEs) and Enterotoxin-like toxins (SEls) responsible for its pathogenic character and are frequently associated to food poisoning and toxic shock syndrome [51]. Indeed, *S. aureus* species is known to produce several exotoxins like staphylokinase, lipases, proteases, collagenases, hemolysins, exfoliative toxins, and superantigen proteins. Many species other than *S. aureus* including *S. cohnii*, *S. epidermis*, *S. xylosus*, *S. haemolyticus*, *S. hyicus,* and *S. intermedius* produce the same virulence factors [52]. This bacterium produces pigmented colonies on Congo red agar and has the ability to colonize, adhere, and form a mature biofilm on both biotic and abiotic surfaces [53,54,55].

The detection of the virulence-related genes in the genome of the identified *S. aureus*, *S. haemolyticus*, and *S. warneri* strains using PCR technique showed the presence of *ica*A gene in 11/19 *S. aureus* strains (57.89%). Similar results have been reported by Arciola et al. [55] suggesting that 60.86% of *S. aureus* strains harbor *ica*A and *ica*D genes. Whereas, Rohde et al. [56] found that all isolated *S. aureus* have the *ica*A gene. Additionally, *ica*A/*ica*D genes were found in the genome of 10/19 *S. aureus* strains (52.63%) including non-slime producing bacteria on CRA. While, Arciola et al. [55] suggested that *ica*A^+^/*ica*D^+^ pattern was detectable only in slime-producing strains.

Seven *S. aureus* strains (36.84%) were positive for the three adhesins genes (*icaD, fnbA,* and *cna*). One *S. Haemolyticus* strain (39B) was positive for two genes (*fnbA*, *cna*). These results were in accordance of those reported by Arciola et al. [57] who reported that 84/191 clinical *S. aureus* strains (44%) harbored two adhesin genes (*fnbA* and *cna*) and only 0.5% of them were positive for *cna* gene. We also noted that 75% of the tested strains were positive for the *fnbA* gene. In fact, Arciola et al. [57] mentioned that 98.4% of *S. aureus* strains isolated from infections associated to implant devices were *fnbA*+ and 99.5% of them were *fnbB*+.

In the present work, the *cna* gene was detected in 54.16% of the identified *Staphylococci*. A previous report demonstrated that the distribution of this gene is related to the area of study and the methodological approach used [58,59]. Additionally, we found that nine isolates were positive for *mec*A gene (37.5%) responsible of methicillin-resistance including one *S. haemolyticus* (7T) and one *S. warneri* strain (30C).

It is well known that a wide range of staphylococcal species harbor the *mec*A gene encoding an alternative penicillin-binding protein 2a [60,61,62,63]. Interestingly, Bouchami et al. [64] reported the amplification of the *mec*A gene in 45 out of 49 (91.8%) clinical bacteremic CoNS isolates (43 *S. epidermidis*, and 2 *S. haemolyticus*) collected in the bone marrow transplant center of Tunisia from 1998 to 2007. In addition, it was demonstrated that the small-size SCCmec types harboring the *mec*A gene are mobile and circulate in the environment leading to the spread of *mec*A and the rise in nosocomial methicillin-resistant staphylococcal infections [65].

In previous works, it was demonstrated that the *mec*A gene is transferred from coagulase-negative staphylococcal species to *S. aureus* in vivo explicating the emergence of more successful *S. aureus* clones with high adherence/invasion capacities [66,67]. In fact, Harisson et al. [68] identified two *Staphylococcus sciuri* subsp. *carnaticus* isolates from bovine infections that harbor three different *mec*A homologues: *mec*A, *mec*A1, and *mec*C. Additionally, a novel allele of *mecA,* namely, *mecC* (originally *mecA*LGA251, with 70% of similitude with *mecA*) was identified in MRSA across Europe from livestock, small mammals, and birds. It was also demonstrated that a circulation of this gene between livestock and humans suggesting a zoonotic reservoir for the human isolates [68,69].

The presence of the *Staphylococci* species isolated on smartphone devices can be explicated by the high incidence of these germs in toilets that can be circulated to the surrounding environment as reported by Mkrtchyan et al. [53]. The same authors founded that 37.8% of these isolates were resistant to antimicrobial agents. Another big issue discussed by Bhoonderowa et al. [54] confirmed that sharing mobile phones among females was associated with high bacterial load.

## 4. Materials and Methods

### 4.1. Sample Collection and Bacteriological Analysis

From February 2017 to May 2017, a survey enrolled 100 students from the High Institute of Biotechnology (Monastir, Tunisia) accepted to be included in the study and gave the consent to analyze their cell phones (*n* = 100). Participants’ ages ranged from 19 to 24 years (M = 20.898, SD = 1.508) with 95% identified as female (*n* = 95) and 5% as male (*n* = 5).

A sterile cotton swab was used to analyze the microflora by drawing horizontal then vertical streaks on the keys or the touch screen, and the cover (plastic, silicone, glass) of the mobile phone. All swabs were kept in brain heart infusion (Biolife, Italy) as transport medium and incubated aerobically at 37 °C for 24 h, then cultured on Chapman (Biolife, Italy) and CHROMagar^TM^ Staph (*CHROMagar* Microbiology, Paris, France) agar Petri dishes at 37 °C for 24 h. Bacterial strains growing on the selective media plates were purified on tryptic soy agar supplemented (TSA, Difco, Spain) plates and subjected to standard morphological (Gram test, catalase, oxidase) biochemical plate or tube tests (coagulase, DNase and mannitol-motility tests). Selected microorganisms were tested for hemolysin on human blood agar (BIO-RAD, France). The enzymes amylase, caseinase, lipase, gelatinase, and lecithinase were detected on media prepared with phosphate buffer saline (PBS) supplemented, respectively, with 0.5% casein peptone, 5% skim milk powder, 1% Tween 80, 5% gelatin powder, and egg yolk emulsion [70]. Strain identification was performed using the MALDI-TOF technique [71].

### 4.2. Susceptibility to Antibiotics

Nineteen antimicrobials agents were tested using the disk diffusion method. A cotton swab was soaked with the bacterial suspension adjusted to 10^7^ CFU/mL (OD_600_ = 0.1) and streaked across the surface of Mueller–Hinton agar medium. Square Petri dishes were dried for five minutes and the following antibiotic disks (Oxoid, UK) were placed on the plates: cefoxitin (FOX, 30 µg), norfloxacin (NOR, 10 µg), fusidic acid (FC, 10 µg), gentamycin (GM, 30 µg), cefotaxim (CTX, 30 µg), chloramphenicol (C, 30 µg), sulfamethoxazole-trimethoprim (SXT, 1.25/23.75 µg), kanamycin (K, 30 µg), tobramycin (TM, 10 µg), tetracycline (TE, 30 µg), erythromycin (E, 15 µg), ofloxacin (OFX, 5 µg), ceftazidime (CAZ, 30 µg), rifampicin (RA, 30 µg), levofloxacin (LEV, 5 µg), amikacin (AN, 30 µg), penicillin G (P, 6 µg), and ticarcillin (TIC, 75 µg). After an overnight incubation at 37 °C for 24 h, the diameters of the zones of inhibition were measured with a 1cm flat ruler.

To interpret the results obtained, we used the multiple antibiotic resistance MAR index defined as a/b, where a represents the number of multiple antibiotics to which the particular isolate is resistant, and b is the number of multiple antibiotics to which the particular isolates were exposed [23,72].

### 4.3. Adhesive Properties of the Identified Bacteria

All identified *Staphylococcus* spp. strains (*n* = 24) were tested qualitatively for their ability to produce exopolysaccharides (Slime) on Congo red agar (CRA) medium and safranin (1%) [73,74]. The bacteria-producing slime on CRA plate gave black colonies with a rough surface against red colonies with a smooth surface for non-producing strains. The safranin technique was conducted according to the technique described for coagulase-negative *Staphylococci*. Briefly, a few colonies of each strain grown on nutrient agar were inoculated into tubes containing 10 mL of Luria Bertani broth supplemented with glucose (final concentration 8%). The tubes were incubated for 24 h at 37 °C and then examined for the presence or absence of membrane formation after the addition of a solution of safranin 1%. The presence of a viscid slime layer was considered as slime producer bacteria. Slime production was scored as negative, weak (+), moderate (++), or strong (+++).

The quantitative analysis of the adhesion ability of the identified *Staphylococcus* spp. strains was tested against two materials that are used for the construction of cell phones: polystyrene and glass using the crystal violet technique [70].

All *Staphylococcus* spp. strains were tested for their ability to form a biofilm on polystyrene 96 well tissue culture plates (Nunc, Roskilde, Denmark) as previously described [75,76]. Two type strains namely *S. aureus* ATCC 25,923 and *S. aureus* ATCC 43,300 were used as positive controls. Biofilm formation was categorized as highly positive (OD_570_ ≥ 1), low-grade positive (0.1 ≤ OD_570_ < 1), or negative (OD_570_ < 0.1). Strips (1.5 cm^2^) were used for the biofilm formation assay on glass material using the protocol described by da Silva Meira [77].

### 4.4. Detection of Methicillin Resistance, Protease (sspA, sspB), Lipase (geh), α-Hemolysin (hla), and Adhesion Genes (icaA, icaD, cna, fnbA) in Staphylococcus spp. Strains

For the bacterial DNA extraction, the inoculated bacterial strains of *S. aureus* were incubating for 18 to 24 h at 37 °C in nutrient broth, pure colonies were suspended in 1 mL of a solution of Tris–EDTA (TE), followed by a centrifugal washing step of this suspension (13,200 rpm, 5 min at 4 °C). Subsequently, the supernatant was removed while the pellet was suspended in a volume of 200 μL TE, vortexed, and then heated at 95 °C for 10 min. After incubation, a final centrifugation step (13,200 rpm, 5 min at 4 °C) was made and the supernatant containing the bacterial DNA was moved into new Eppendorf tubes. The DNA was stored at −20 °C until use.

The detection of the *mec*A gene responsible for methicillin resistance was carried out according to the technique described by Geha et al. [78]. The presence of protease (*sspA*, *sspB*), lipase (*geh*), β-hemolysin (*hla*), and adhesion (*icaA, icaD, can, fnbA*) genes in the genome of the tested strains was also detected by using the protocol described by Karlsson et al. [79]. The *hla* gene was detected using the protocol described by Liang et al. [59]. All primers used, PCR conditions, and amplicon size are reported in Table 7.

### 4.5. Data Analysis

Phylogenetic trees based on phenotypic and/or genetic traits were constructed using the unweighted pair group method of arithmetic averages (UPGMAs) and Jaccard’s correlation coefficient [82]. The estimation of biofilm capacity formation on polystyrene and glass surfaces was conducted in triplicates for each isolate. Mean and standard deviation values were performed with SPSS 17.0 software. The non-parametric Mann–Whitney U test was used to correlate between the quantitative biofilm production assays and the distribution of (exoenzymes/biofilm/hemolysin) related genes.

## 5. Conclusions

Our study demonstrably highlighted that many *Staphylococci* species can be associated with smartphones of students. Most of these bacteria produce several virulence-related factors that allow them to adhere and form a mature biofilm on these devices. Thus, probably allowing to them to be transferred to the organs coming in deep contact with the cell phone. Interestingly, the *mec*A gene, responsible for resistance to methicillin was amplified in two coagulase-negative *Staphylococcus* species (*S. haemolyticus* and *S. warneri*) leading to the emergence of new clones of drug resistant bacteria. The lack of awareness of using cell phones may contribute to a significant risk of transmitting multidrug-resistant bacteria through unguarded cell phone use. New biomaterial used for smartphone manufacturing or decontamination solution have to be designed to avoid the contamination by these bacteria.

## Figures and Tables

**Table 1:** Morphotypes of *Staphylococcus* spp. strains isolated from CHROM^TM^ Staph agar based on the color scale.

Organism (No. Tested)	Color of Isolated Colonies
Methicillin susceptible (1)	Mauve
Methicillin resistant (18)	Mauve
*S. haemolyticus* (2)	Light blue
*S. warneri* (3)	White

**Table 2:** Hydrolytic enzymes production and MALDI-TOF identification.

Strains	MALDI-TOF-MS	1	2	3	4	5	6	7	8	9
ATCC 43300	*S. aureus*	+	+	+	+	−	+	+	+	β
12C	*S. aureus*	+	+	+	+	−	+	−	+	β
26M	*S. aureus*	+	+	+	+	+	+	+	+	β
32T	*S. aureus*	+	+	+	+	+	−	+	−	α
34B	*S. aureus*	+	+	+	+	−	−	+	+	α
37C	*S. aureus*	+	+	+	+	−	−	+	−	α
40B	*S. aureus*	+	+	+	−	+	+	+	+	α
47C	*S. aureus*	+	+	+	−	+	−	+	−	β
56M	*S. aureus*	+	+	+	+	−	−	+	−	α
57 M	*S. aureus*	+	+	+	+	−	−	+	−	α
59B	*S. aureus*	+	+	+	+	+	−	+	−	σ
65C	*S. aureus*	+	+	+	+	+	−	−	−	σ
66B	*S. aureus*	+	+	+	−	−	−	+	−	α
67M	*S. aureus*	+	+	+	−	−	+	+	+	β
68T	*S. aureus*	+	+	+	−	−	+	+	−	β
74M	*S. aureus*	+	+	+	+	+	+	+	−	α
76C	*S. aureus*	+	+	+	+	−	−	+	+	β
78T	*S. aureus*	+	+	+	+	+	−	+	−	σ
83M	*S. aureus*	+	+	+	+	+	+	+	+	β
96M	*S. aureus*	+	+	+	+	+	+	+	−	σ
7T	*S. warneri*	−	+	+	+	−	−	−	−	α
31C	*S. warneri*	−	+	+	+	−	+	−	−	α
47B	*S. warneri*	−	+	+	−	−	+	+	−	σ
30C	*S. haemolyticus*	−	+	+	+	+	+	+	−	α
39B	*S. haemolyticus*	−	+	+	−	−	−	+	−	α
**% positivity**		**80**	**100**	**100**	**70.83**	**45.83**	**45.83**	**83.33**	**29.16**	**29.16 (β)**

Note: 1—Coagulase; 2—DNase; 3—Catalase; 4—Amylase; 5—Caseinase; 6—Gelatinase; 7—Lecithinase; 8—Lipase; 9—Hemolysins.

**Table 3:** Susceptibility profiles of *Staphylococcus* spp. isolated from cell phone mobiles and PCR-detection of *mec*A gene.

Strains	Antibiotics																		MARI	*mec*A
	1	2	3	4	5	6	7	8	9	10	11	12	13	14	15	16	17	18		
ATCC 43300	R	S	R	R	R	R	R	S	S	R	S	S	R	R	S	S	R	R	0.611	mecA+
12C	R	S	S	R	R	S	R	S	R	R	R	S	R	R	S	R	R	R	0.666	mecA−
26M	R	S	S	S	R	S	R	S	R	R	S	S	R	S	S	S	R	R	0.444	mecA+
32T	R	S	R	S	R	S	R	S	R	R	R	S	R	R	S	S	R	R	0.611	mecA−
34B	R	S	R	S	R	S	S	S	R	S	R	S	R	S	S	S	R	S	0.388	mecA−
37C	R	S	R	S	R	S	R	S	R	R	R	S	R	S	S	S	R	R	0.555	mecA−
40B	R	S	R	S	R	S	S	S	S	S	R	S	R	R	S	S	R	R	0.444	mecA−
47C	R	S	R	S	R	S	S	S	R	R	R	S	R	R	S	S	R	R	0.555	mecA−
56M	S	S	R	R	R	S	R	S	R	R	S	S	R	R	S	S	R	R	0.555	mecA−
57M	R	S	R	R	R	R	S	S	R	R	R	S	R	S	S	R	R	R	0.666	mecA−
59B	R	S	R	R	R	R	R	R	R	R	R	S	R	S	S	R	R	R	0.777	mecA+
65C	R	S	S	S	R	S	S	S	S	R	S	S	R	R	S	S	R	R	0.388	mecA+
67M	R	S	R	R	R	S	R	S	R	R	R	S	R	R	S	S	R	R	0.666	mecA−
66B	R	S	S	S	R	S	S	R	R	R	R	S	R	S	S	R	R	R	0.555	mecA+
68T	R	R	R	R	-	S	-	S	R	R	R	R	R	S	R	R	R	R	0.812	mecA+
74M	R	S	R	S	R	R	R	S	S	R	S	R	R	R	S	S	R	R	0.611	mecA+
76C	R	S	S	S	R	S	R	S	R	S	R	S	R	R	S	S	R	R	0.500	mecA−
78T	R	S	R	S	R	S	R	S	S	R	R	S	R	R	S	S	R	R	0.555	mecA+
83M	R	S	S	S	R	S	R	S	S	R	R	S	R	R	R	S	R	R	0.555	mecA−
96M	R	S	R	R	R	R	R	S	R	R	R	S	R	R	S	S	R	R	0.722	mecA−
7T	R	R	S	R	R	S	R	S	R	R	R	S	R	R	S	R	R	R	0.722	mecA+
31C	R	S	R	R	R	R	R	R	R	R	R	S	R	S	S	R	R	R	0.777	mecA−
47B	R	S	S	S	R	R	-	S	R	R	S	S	R	R	S	S	R	R	0.529	mecA−
39B	R	S	R	R	R	S	R	S	R	R	R	R	R	R	S	S	R	R	0.722	mecA+
30C	R	S	S	R	R	S	R	S	R	R	R	S	R	R	S	R	R	R	0.666	mecA−

Note: Antibiotics are listed from 1 to 18; cefotaxim (CTX); penicillin G (P); ceftazidim (CAZ); cefoxitin (FOX); ticarcillin (TIC); tetracyclin (TE); erythromycin (E); tobramycin (TM); sulfamethoxazole-Trimethoprim (SXT); rifampicin (RA); fusidic acid (FC); gentamicin (GM); amikacin (AN); chloramphenicol (C); kanamycin (K); ofloxacin (OFX); norfloxacin (NOR); levofloxacin (LEV); -: non tested; MARI: Multiple Antibiotic Resistance Index.

**Table 4:** Exopolysaccharide production (CRA), glass adhesion (Safranin 1%), and biofilm formation on polystyrene and glass by *Staphylococcus* spp. strains.

Strains	Safranin Assay	Slime Phenotype (CRA)		Biofilm on Polystyrene		Biofilm on Glass	
				(OD_570_) ± SD	Interpretation	(OD_570_) ± SD	Interpretation
ATCC 43300	+++	Black	S+	1.89 ± 0.13	H	1.48 ± 0.15	H
12C	+++	Black	S+	1.34 ± 0.18	H	1.09 ± 0.05	H
26M	+++	Red	S−	1.31 ± 0.15	H	0.57 ± 0.1	M
32T	+	Bordeaux	S−	0.57 ± 0.4	M	0.55 ± 0.04	M
34B	+++	Red	S−	0.64 ± 0	M	0.65 ± 0.09	M
37C	++	Red	S−	0.16 ± 0.35	M	0.99 ± 0.01	M
40B	+++	Bordeaux	S−	0.28 ± 0	M	1.10 ± 0.16	H
47C	++	Red	S−	0.64 ± 0	M	0.57 ± 0.06	M
56M	++	Red with black center	S+	0.21 ± 0	M	1.11 ± 0.08	H
57 M	+++	Red	S−	0.74 ± 0	M	1.68 ± 0.2	H
59B	+++	Pink	S−	0.16 ± 0	M	0.717 ± 0.12	M
65C	++	Pink	S−	0.15 ± 0	M	0.88 ± 0.05	M
66B	++	Red	S−	2.73 ± 0.56	H	0.75 ± 0.2	M
67M	+++	Orange	S−	1.27 ± 0.17	H	1.28 ± 0.1	H
68T	+++	Red	S−	2.14 ± 0.64	H	0.45 ± 0.09	M
74M	+++	Pink	S−	0.40 ± 0	M	0.50 ± 0.09	M
76C	+++	Black	S+	1.63 ± 0.57	H	0.38 ± 0.04	M
78T	+	Red	S−	0.47 ± 0	M	0.90 ± 0.15	M
83M	+++	Red with black center	S+	0.85 ± 0.13	M	0.32 ± 0.04	M
96M	+++	Red	S−	0.36 ± 0.3	M	0.30 ± 0.05	M
7T	+++	Red	S−	0.33 ± 0	M	0.87 ± 0.13	M
31C	+++	Pink	S−	0.59 ± 0	M	0.46 ± 0.08	M
47B	++	Pink	S−	0.66 ± 0.12	M	0.56 ± 0.03	M
30C	+	Red	S−	0.17 ± 0	M	0.98 ± 0.07	M
39B	++	Pink	S−	1.07 ± 0.19	H	0.84 ± 0.11	M

Note: CRA—Congo red agar; OD_570nm_—optical density at 570 nm; SD—standard deviation; H—high biofilm; M—moderate biofilm; S+—slime producer; S−—non-slime producer; Slime production by safranin technique was scored as negative, weak (+), moderate (++), or strong (+++).

**Table 5:** Distribution of biofilm related genes (*icaA*, *icaD*, *cna*, *fnbA*), α-hemolysin (*hla*), and exoenzymes genes (*geh*, *sspA*, *sspB*) in *Staphylococcus* spp. strains genome.

Strains	Adhesion				Haemolysins	Exoenymes		
	*icaA*	*icaD*	*cna*	*fnbA*	*Hla*	*geh*	*sspA*	*sspB*
ATCC 43300	+	+	+	+	+	+	+	+
12C	−	−	−	+	−	+	−	+
26M	−	−	+	+	+	+	−	+
32T	+	+	−	+	+	+	−	−
34B	+	+	+	+	−	+	−	+
37C	+	+	+	+	−	−	−	+
40B	−	−	−	−	−	−	−	+
47C	−	+	+	−	+	−	+	+
56M	+	+	+	+	+	+	−	+
57M	+	+	−	+	−	+	−	+
59B	−	+	+	−	+	−	+	+
65C	−	−	+	+	+	−	−	+
66B	+	−	−	+	−	−	−	+
67M	+	+	+	−	+	−	−	−
68T	+	+	+	+	+	+	−	+
74M	+	+	+	+	−	−	−	−
76C	+	+	+	+	−	−	−	+
78T	+	+	+	+	−	−	−	+
83M	+	−	−	+	−	−	−	−
96M	+	−	−	+	+	−	−	−
7T	−	+	−	−	−	−	−	−
31C	−	+	−	−	−	−	−	+
47B	−	+	−	+	−	−	−	−
30C	+	+	−	+	−	−	+	−
39B	+	−	+	+	−	+	+	+
**% positivity**	**62.5**	**66.66**	**50.00**	**75.00**	**37.50**	**33.33**	**16.66**	**66.66**

**Table 6:** Correlation between the ability to form a biofilm on polystyrene and glass material with biofilm related genes (*icaA*, *icaD*, *cna*, *fnbA*), α-hemolysin (*hla*), and exoenzymes genes (*geh*, *sspA*, *sspB*).

*p*-Value (Univariate Analysis)	Adhesion Related Genes				Haemolysin Gene	Exoenymes Genes		
	*icaA*	*icaD*	*cna*	*fnbA*	*Hla*	*geh*	*sspA*	*sspB*
Biofilm on glass	0.489	0.783	0.783	0.489	0.581	0.945	0.836	0.581
Biofilm on polystyrene	0.489	0.783	0.783	0.489	0.581	0.945	0.836	0.581

**Table 7:** Selected specific primers and PCR-amplification conditions.

Primer	Primer Sequence	PCR Conditions	Product Size (pb)	Reference
*mecA*-F	5′-GTA GAA ATG ACT GAA CGT CCG ATAA-3′	94 °C for 5 min 30 Cycles (1 min at 94 °C, 1 min at 55 °C, 2 min at 72 °C) 72 °C for 10 min	310	[78]
*mecA*-R	5′-CCAATT CCA CAT TGT TTC GGT CTAA-3′			
*sspA*-F	5′-GAC AAC AGC GAC ACT TGT GA-3′	94 °C for 5 min 30 Cycles (30 s at 94 °C, 30 s min at 45 °C, 45 s at 72 °C) 72 °C for 10 min	292	[79]
*sspA*-R	5′-AGT ATC TTT ACC TAC AAC TAC A-3′			
*sspB*-F	5′-TGA AGA AGA TGG CAA AGT TAG-3′	94 °C for 5 min 30 Cycles (30 s at 94 °C, 30 s min at 47 °C, 45 s at 72 °C) 72 °C for 10 min	493	
*sspB*-R	5′-TTG AGA TAC ACT TTG TGC AAG-3′			
*geh*-F	5′-GCACAAGCCTCGG-3′	94 °C for 5 min 30 Cycles (30 s at 94 °C, 30 s min at 40 °C, 45 s at 72 °C) 72 °C for 10 min	473	[20]
*geh*-R	5′-GACGGGGGTGTAG-3′			
*icaA*-F	5′-ACACTTGCTGGCGCAGTCAA-3′	94 °C for 5 min 30 Cycles (30 s at 94 °C, 30 s min at 45 °C, 45 s at 72 °C) 72 °C for 10 min	188	[80]
*icaA*-R	5′-TCTGGAACCAACATCCAACA-3′			
*icaD*-F	5′-ACACTTGCTGGCGCAGTCAA-3′	94 °C for 5 min 30 Cycles (30 s at 94 °C, 30 s min at 55 °C, 45 s at 72 °C) 72 °C for 10 min	198	
*icaD*-R	5′-TCTGGAACCAACATCCAACA-3′			
*cna*-F	5′-AAAGCGTTGCCTAGTGGAGA-3′	94 °C for 5 min 30 Cycles (30 s at 94 °C, 30 s min at 62 °C, 45 s at 72 °C) 72 °C for 10 min	192	[57]
*cna*-R	5′-AGTGCCTTCCCAAACCTTTT-3′			
*fnb*A-F	5′-GATACAAACCCAGGTGGTGG-3′		191	
*fnb*A-R	5′-TGTGCTTGACCATGCTCTTC-3′			
*hla*-F	5′CAACTGATAAAAAAGTAGGCTGGAAAGTGAT-3′	94 °C for 5 min 35 Cycles (30 s at 94 °C, 60 s min at 59 °C, 60 s at 72 °C) 72 °C for 10 min	201	[81]
*hla*-R	5′-CTGGTGAAAACCCTGAAGATAATAGAG-3′			

## References

[1] Halayem S., Nouira O., Bourgou S., Bouden A., Othman S., Halayem M. (2010). The mobile: A new addiction upon adolescents. Tunis. Med..

[2] Schabrun S.M., Hoorn W.V.D., Moorcroft A., Greenland C., Hodges P.W. (2014). Texting and Walking: Strategies for Postural Control and Implications for Safety. PLoS ONE.

[3] Hamilton J. (2003). Are main lines and mobile phones substitutes or complements? Evidence from Africa. Telecommun. Policy.

[4] Laatar R., Kachouri H., Borji R., Rebai H., Sahli S. (2017). The effect of cell phone use on postural balance and mobility in older compared to young adults. Physiol. Behav..

[5] Ekrakene T., Igeleke C.L. (2007). Microorganisms Associated with Public Mobile Phones Along Benin-Sapele express way. J. Appl. Sci. Res..

[6] Bhat S.S., Hegde S.K., Salian S. (2011). Potential of Mobile Phones to Serve as a Reservoir in Spread of Nosocomial Pathogens. Online J. Health Allied Sci..

[7] Egert M., Späth K., Weik K., Kunzelmann H., Horn C., Kohl M., Blessing F. (2014). Bacteria on smartphone touchscreens in a German university setting and evaluation of two popular cleaning methods using commercially available cleaning products. Folia Microbiol..

[8] Pierson D.J. (2013). Is your smart phone spreading infection?. Int. Med. Alert.

[9] Brady R.R., Verran J., Damani N., Gibb A. (2009). Review of mobile communication devices as potential reservoirs of nosocomial pathogens. J. Hosp. Infect..

[10] Brady R., Wasson A., Stirling I., McAllister C., Damani N. (2006). Is your phone bugged? The incidence of bacteria known to cause nosocomial infection on healthcare workers’ mobile phones. J. Hosp. Infect..

[11] Gil Goldblatt J., Krief I., Klonsky T., Haller D., Milloul V., Sixsmith D.M., Srugo I., Potasman I. (2007). Use of Cellular Telephones and Transmission of Pathogens by Medical Staff in New York and Israel. Infect. Control Hosp. Epidemiol..

[12] Onyango L., Alreshidi M.M. (2018). Adaptive Metabolism in Staphylococci: Survival and Persistence in Environmental and Clinical Settings. J. Pathog..

[13] Alreshidi M.M., Dunstan R.H., Onyango L.A., Roberts T.K., Mendez-Vilas A. (2013). “Staphylococcal phenomics: Metabolomic and proteomic responses to environmental stressors”. Microbial Pathogens and Strategies for Combating Them: Science, Technology and Education.

[14] Alreshidi M.M., Dunstan R.H., Macdonald M.M., Smith N.D., Gottfries J., Roberts T.K., Gottries J. (2015). Metabolomic and proteomic responses of *Staphylococcus aureus* to prolonged cold stress. J. Proteom..

[15] Alreshidi M.M., Dunstan R.H., Gottfries J., Macdonald M.M., Crompton M.J., Ang C.-S., Williamson N.A., Roberts T.K. (2016). Changes in the Cytoplasmic Composition of Amino Acids and Proteins Observed in *Staphylococcus aureus* during Growth under Variable Growth Conditions Representative of the Human Wound Site. PLoS ONE.

[16] Onyango L., Dunstan R.H., Gottfries J., Von Eiff C., Roberts T.K. (2012). Effect of Low Temperature on Growth and Ultra-Structure of *Staphylococcus* spp.. PLoS ONE.

[17] Vuong C., Kocianova S., Voyich J.M., Yao Y., Fischer E.R., DeLeo F.R., Otto M. (2004). A Crucial Role for Exopolysaccharide Modification in Bacterial Biofilm Formation, Immune Evasion, and Virulence. J. Biol. Chem..

[18] Kanayama A.K., Takahashi H., Yoshizawa S., Tateda K., Kaneko A., Kobayashi I. (2017). *Staphylococcus aureus* surface contamination of mobile phones and presence of genetically identical strains on the hands of nursing personnel. Am. J. Infect. Control.

[19] Foster T.J., Höök M. (1998). Surface protein adhesins of *Staphylococcus aureus*. Trends Microbiol..

[20] Saïd-Salim B., Dunman P.M., McAleese F.M., Macapagal D., Murphy E., McNamara P.J., Arvidson S., Foster T.J., Projan S.J., Kreiswirth B.N. (2003). Global Regulation of *Staphylococcus aureus* Genes by Rot. J. Bacteriol..

[21] Lowy F.D. (1998). *Staphylococcus aureus* infections. N. Engl. J. Med..

[22] Deresse D., Daka D. (2014). Antibiotic-resistant *Staphylococcus aureus* isolated from mobile phone and hands of Health care workers in the Hawassa referral Hospital, South Ethiopia. J. Microbiol. Antimicrob..

[23] Krumperman P.H. (1983). Multiple antibiotic resistance indexing of *Escherichia coli* to identify high-risk sources of fecal contamination of foods. Appl. Environ. Microbiol..

[24] Srikanth P., Ezhil R., Suchitra S., Anandhi I., Maheswari U., Kalyani J. (2008). The Mobile Phone in a Tropical Setting—Emerging Threat for Infection Control. Int. J. Infect. Dis..

[25] Mark D., Leonard C., Breen H., Graydon R., O’Gorman C., Kirk S. (2014). Mobile phones in clinical practice: Reducing the risk of bacterial contamination. Int. J. Clin. Pract..

[26] Tagoe D.N., Gyande V.K., Ansah E.O. (2011). Bacterial contamination of mobile phones: When your mobile phone could transmit more than just a call. WebmedCentral.

[27] Datta P., Rani H., Chander J., Gupta V. (2009). Bacterial contamination of mobile phones of health care workers. Indian J. Med. Microbiol..

[28] Ulger F., Essen S., Dilek A., Yanik K., Gunaydin M., Leblebicioglu H. (2009). Are we aware how contaminated our mobile phones are with nosocomial pathogens?. Ann. Clin. Microb. Antimirob..

[29] Badr R.I., Badr H.I., Ali N.M. (2012). Mobile phones and nosocomial infections. Int. J. Infect. Control.

[30] Lavanya J., Rani N.S., Jais M., Upadhya A.K. (2016). Microbial Contamination of Mobile Phones in a Tertiary Health Care Setting. Int. J. Curr. Microbiol. Appl. Sci..

[31] Trivedi H.R., Desai K.J., Trivedi L.P., Malek S.S., Javdekar T. (2011). Role of Mobile Phone in Spreading Hospital Acquired Infection: A Study in Different Group of Health Care Workers. Natl. J. Integr. Res. Med..

[32] Pal K., Chatterjee M., Sen P., Adhya S. (2015). Cell Phones of Health Care Professionals: A Silent Source of Bacteria. Natl. J. Lab. Med..

[33] Tekerekoǧlu M.S., Duman Y., Serindag A., Cuǧlan S.S., Kaysadu H., Tunc E., Yakupogullari Y., Tekerekoglu M.S. (2011). Do mobile phones of patients, companions and visitors carry multidrug-resistant hospital pathogens?. Am. J. Infect. Control.

[34] Yu F., Li T., Huang X., Xie J., Xu Y., Tu J., Qin Z., Parsons C., Wang J., Hu L. (2012). Virulence gene profiling and molecular characterization of hospital-acquired *Staphylococcus aureus* isolates associated with bloodstream infection. Diagn. Microbiol. Infect. Dis..

[35] Vorobieva V., Bazhukova T., Hanssen A.M., Caugant D.A., Semenova N., Haldorsen B.C., Simonsen G.S., Sundsfjord A. (2008). Clinical isolates of *Staphylococcus aureus* from the Arkhangelsk region, Russia: Antimicrobial susceptibility, molecular epidemiology, and distribution of panton-valentine leukocidin genes. APMIS.

[36] Teixeira M.M., Araujo M., Silva-Carvalho M., Beltrame C.O., Oliveira C., Figueiredo A., Oliveira A. (2012). Emergence of clonal complex 5 (CC5) methicillin-resistant *Staphylococcus aureus* (MRSA) isolates susceptible to trimethoprim-sulfamethoxazole in a Brazilian hospital. Braz. J. Med. Biol. Res..

[37] Maple P.A.C., Hamilton-Miller J., Brumfitt W. (1989). World-wide antibiotic resistance in methicillin-resistant *Staphylococcus aureus*. Lancet.

[38] Nimmo G.R., Bell J.M., Mitchell D., Gosbell I., Pearman J.W., Turnidge J.D. (2003). Antimicrobial Resistance in *Staphylococcus aureus* in Australian Teaching Hospitals, 1989–1999. Microb. Drug Resist..

[39] Tokajian S., Haddad M., Andraos R., Hashwa F., Araj G. (2011). Toxins and Antibiotic Resistance in *Staphylococcus aureus* Isolated from a Major Hospital in Lebanon. ISRN Microbiol..

[40] Hamze M., Dabboussi F., Daher W., Izard D. (2003). Antibiotic resistance of *Staphylococcus aureus* at north Lebanon: Place of the methicillin resistance and comparison of detection methods. Pathol. Biol..

[41] Bischoff W.E., Wallis M.L., Tucker K.B., Reboussin B.A., Sherertz R.J. (2004). *Staphylococcus aureus* Nasal Carriage in a Student Community Prevalence, Clonal Relationships, and Risk Factors. Infect. Control Hosp. Epidemiol..

[42] Tambe N.N., Pai C. (2012). A Study of microbial flora and MRSA harboured by mobile phones of health care personnel. Int. J. Recent Trends Sci. Technol..

[43] Morubagal R.R., Shivappa S.G., Mahale R.P., Neelambike S.M. (2017). Study of bacterial flora associated with mobile phones of healthcare workers and non-healthcare workers. Iran J. Microbiol..

[44] Safdari H., Aryan E., Sadeghian H., Shams S.F., Aganj M. (2020). Frequency of methicillin-resistant *Staphylococcus aureus* (MRSA) in nose and cellular phone of medical and non-medical personnel of emergency departments of Ghaem hospital in Mashhad city. Clin. Epidemiol. Glob. Health..

[45] Wu P.Z., Zhu H., Thakur A., Willcox M.D. (1999). Comparison of potential pathogenic traits of staphylococci that may contribute to corneal ulceration and inflammation. Aust. N.-Z. J. Ophthalmol..

[46] Barretti P., Montelli A.C., Batalha J.E., Caramori J.C., Cunha M.L. (2009). The role of virulence factors in the outcome of staphylococcal peritonitis in CAPD patients. BMC Infect. Dis..

[47] Kouidhi B., Zmantar T., Hentati H., Bakhrouf A. (2010). Cell surface hydrophobicity, biofilm formation, adhesives properties and molecular detection of adhesins genes in *Staphylococcus aureus* associated to dental caries. Microb. Pathog..

[48] Khoramian B., Jabalameli F., Niasari-Naslaji A., Taherikalani M., Emaneini M. (2015). Comparison of virulence factors and biofilm formation among *Staphylococcus aureus* strains isolated from human and bovine infections. Microb. Pathog..

[49] Zmantar T., Chaieb K., Makni H., Miladi H., Ben Abdallah F., Mahdouani K., Bakhrouf A. (2008). Detection by PCR of adhesins genes and slime production in clinical *Staphylococcus aureus*. J. Basic Microbiol..

[50] Haddad O., Merghni A., Elargoubi A., Rhim H., Kadri Y., Mastouri M. (2018). Comparative study of virulence factors among methicillin resistant *Staphylococcus aureus* clinical isolates. BMC Infect. Dis..

[51] Pinchuk I.V., Beswick E.J., Reyes V.E. (2010). Staphylococcal enterotoxins. Toxins (Basel).

[52] Benkerroum N. (2017). Staphylococcal enterotoxins and enterotoxin-like toxins with special reference to dairy products: An overview. Crit. Rev. Food Sci. Nutr..

[53] Mkrtchyan H.V., Russell C.A., Wang N., Cutler R.R. (2013). Could Public Restrooms Be an Environment for Bacterial Resistomes?. PLoS ONE.

[54] Bhoonderowa A., Gookool S., Biranjia-Hurdoyal S.D., Biranjia-Hurdoyal S. (2014). The Importance of Mobile Phones in the Possible Transmission of Bacterial Infections in the Community. J. Community Health.

[55] Arciola C.R., Baldassarri L., Montanaro L. (2001). Presence of icaA and icaD Genes and Slime Production in a Collection of Staphylococcal Strains from Catheter-Associated Infections. J. Clin. Microbiol..

[56] Rohde H., Knobloch J., Horstkotte M.A., Mack D. (2001). Correlation of *Staphylococcus aureus* icaADBC genotype and biofilm expression phenotype. J. Clin. Microbiol..

[57] Arciola C.R., Campoccia D., Gamberini S., Baldassarri L., Montanaro L. (2005). Prevalence of cna, fnbA and fnbB adhesin genes among *Staphylococcus aureus* isolates from orthopedic infections associated to different types of implant. FEMS Microbiol. Lett..

[58] Thomas M.G., Peacock S., Daenke S., Berendt A.R., Young B., Johnson S., Minoo B., Shugarts D., Allen M., Ramey R.R. (1999). Adhesion of *Staphylococcus aureus* to Collagen Is Not a Major Virulence Determinant for Septic Arthritis, Osteomyelitis, or Endocarditis. J. Infect. Dis..

[59] Peacock S.J., Moore C.E., Justice A., Kantzanou M., Story L., Mackie K., O’Neill G., Day N.P.J. (2002). Virulent Combinations of Adhesin and Toxin Genes in Natural Populations of *Staphylococcus aureus*. Infect. Immun..

[60] Hartman B.J., Tomasz A. (1984). Low-affinity penicillin-binding protein associated with β-lactam resistance in *Staphylococcus aureus*. J. Bacteriol..

[61] Ubukata K., Nonoguchi R., Matsuhashi M., Konno M. (1989). Expression and inducibility in *Staphylococcus aureus* of the mecA gene, which encodes a methicillin-resistant *S. aureus*-specific penicillin-binding protein. J. Bacteriol..

[62] Lim D., Strynadka N.C. (2002). Structural basis for the β-lactam resistance of PBP2a from methicillin-resistant *Staphylococcus aureus*. Nat. Struct. Biol..

[63] Fuda C., Suvorov M., Vakulenko S.B., Mobashery S. (2004). The Basis for Resistance to β-Lactam Antibiotics by Penicillin-binding Protein 2a of Methicillin-resistant *Staphylococcus aureus*. J. Biol. Chem..

[64] Bouchami O., Achour W., Mekni M.A., Rolo J., Ben Hassen A. (2011). Antibiotic resistance and molecular characterization of clinical isolates of methicillin-resistant coagulase-negative staphylococci isolated from bacteremic patients in oncohematology. Folia Microbiol..

[65] Noto M.J., Archer G.L. (2006). A Subset of *Staphylococcus aureus* Strains Harboring Staphylococcal Cassette Chromosome mec (SCCmec) Type IV Is Deficient in CcrAB-Mediated SCCmec Excision. Antimicrob. Agents Chemother..

[66] Harrison E.M., Paterson G.K., Holden M.T.G., Ba X., Rolo J., Morgan F.J.E., Pichon B., Kearns A., Zadoks R.N., Peacock S.J. (2013). A novel hybrid SCCmec-mecC region in *Staphylococcus sciuri*. J. Antimicrob. Chemother..

[67] Szczuka E., Krzyminska S., Bogucka N., Kaznowski A. (2017). Multifactorial mechanisms of the pathogenesis of methicillin-resistant *Staphylococcus hominis* isolated from bloodstream infections. Antonie Van Leeuwenhoek.

[68] Harrison E.M., Paterson G.K., Holden M.T.G., Larsen J., Stegger M., Larsen A.R., Petersen A., Skov R.L., Christensen J.M., Zeuthen A.B. (2013). Whole genome sequencing identifies zoonotic transmission of MRSA isolates with the novel mecA homologue mecC. EMBO Mol. Med..

[69] Petersen A., Stegger M., Heltberg O., Christensen J., Zeuthen A., Knudsen L., Urth T., Sorum M., Schouls L., Larsen J. (2013). Epidemiology of methicillin-resistant *Staphylococcus aureus* carrying the novel mecC gene in Denmark corroborates a zoonotic reservoir with transmission to humans. Clin. Microbiol. Infect..

[70] Snoussi M., Noumi E., Hajlaoui H., Usai D., Sechi L.A., Zanetti S., Bakhrouf A. (2009). High potential of adhesion to abiotic and biotic materials in fish aquaculture facility by *Vibrio alginolyticus* strains. J. Appl. Microbiol..

[71] Eddouzi J., Hofstetter V., Groenewald M., Manai M., Sanglard D. (2012). Characterization of a New Clinical Yeast Species, *Candida tunisiensis* sp. nov., Isolated from a Strain Collection from Tunisian Hospitals. J. Clin. Microbiol..

[72] Manjusha S., Sarita G., Elyas K., Chandrasekaran M. (2005). Multiple Antibiotic Resistances of *Vibrio* Isolates from Coastal and Brackish Water Areas. Am. J. Biochem. Biotechnol..

[73] Christensen G.D., Simpson W.A., Bisno A.L., Beachey E.H. (1982). Adherence of biofilm producing strains of *Staphylococcus epidermidis* to smooth surfaces. Infect. Immun..

[74] Freeman D.J., Falkiner F.R., Keane C.T. (1989). New method for detecting slime production by coagulase negative staphylococci. J. Clin. Pathol..

[75] Mack D., Bartscht K., Fischer C., Rohde H., De Grahl C., Dobinsky S., Horstkotte M.A., Kiel K., Knobloch J.K. (2001). Genetic and biochemical analysis of *Staphylococcus epidermidis* biofilm accumulation. Methods Enzymol..

[76] Rachid S., Ohlsen K., Wallner U., Hacker J., Hecker M., Ziebuhr W. (2000). Alternative Transcription Factor ςB Is Involved in Regulation of Biofilm Expression in a *Staphylococcus aureus* Mucosal Isolate. J. Bacteriol..

[77] da Silva Meira Q.G., de Medeiros Barbosa I., Alves Aguiar Athayde A.J., de Siqueira-Júnior J.P., de Souza E.L. (2012). Influence of temperature and surface kind on biofilm formation by *Staphylococcus aureus* from food-contact surfaces and sensitivity to sanitizers. Food Control.

[78] Geha D.J., Uhl J.R., Gustaferro C.A., Persing D.H. (1994). Multiplex PCR for identification of methicillin-resistant staphylococci in the clinical laboratory. J. Clin. Microbiol..

[79] Karlsson A., Arvidson S. (2002). Variation in Extracellular Protease Production among Clinical Isolates of Staphylococcus aureus Due to Different Levels of Expression of the Protease Repressor sarA. Infect. Immun..

[80] Arciola C.R., Borsetti E., Collamati S., Baldassarri L., Montanaro L. (1999). Detection of fibronectin-binding protein genes in staphylococcal strains from peri-prosthesis infections. New Microbiol..

[81] Liang X., Ji Y. (2006). Alpha-toxin interferes with integrin-mediated adhesion and internalization of *Staphylococcus aureus* by epithelial cells. Cell. Microbiol..

[82] Snoussi M., Noumi E., Usai D., Sechi L.A., Zanetti S., Bakhrouf A. (2008). Distribution of some virulence related-properties of *Vibrio alginolyticus* strains isolated from Mediterranean seawater (Bay of Khenis, Tunisia): Investigation of eight *Vibrio cholerae* virulence genes. World J. Microbiol. Biotechnol..

